# Phospholipid remodeling as a key regulator of ferroptosis

**DOI:** 10.1016/j.jlr.2026.101091

**Published:** 2026-06-25

**Authors:** Tomoyoshi Shingaki, Junken Aoki, Nozomu Kono

**Affiliations:** Laboratory of Health Chemistry, Graduate School of Pharmaceutical Sciences, the University of Tokyo, Tokyo, Japan

**Keywords:** ferroptosis, phospholipid, phospholipid remodeling, lysophospholipid acyltransferase, acyl-CoA synthetase, phospholipase A

## Abstract

Ferroptosis is an iron-dependent form of regulated cell death characterized by the accumulation of lipid peroxides in cellular membranes. Cellular susceptibility to ferroptosis is strongly influenced by membrane phospholipid composition, which is dynamically regulated through phospholipid remodeling. Phospholipid remodeling, also known as the Lands’ cycle, drives the replacement of fatty acyl chains in phospholipids through the coordinated actions of phospholipases A, acyl-CoA synthetases (ACSLs), and lysophospholipid acyltransferases (LPLATs). Phospholipid remodeling critically influences ferroptosis sensitivity by regulating the balance between phospholipid species containing polyunsaturated fatty acids (PUFAs), which promote lipid peroxidation, and those containing saturated/monounsaturated fatty acids, which confer resistance. Recent studies have identified key remodeling enzymes, including ACSL4 and LPLAT12, as central drivers of ferroptosis through the generation of PUFA-containing phospholipids, while other enzymes suppress ferroptosis by limiting lipid peroxidation or removing oxidized phospholipids. In parallel, specific phospholipid species—including arachidonic acid- and adrenic acid-containing phospholipids, di-PUFA phospholipids, and other oxidizable lipid classes—have emerged as critical contributors to ferroptosis. Collectively, these findings highlight phospholipid remodeling as a central determinant of ferroptosis by shaping the membrane lipid landscape.

Ferroptosis is a regulated form of cell death characterized by the iron-dependent accumulation of lipid peroxides within cellular membranes. Since its original description in 2012 (1), ferroptosis has been recognized as a mechanistically distinct mode of cell death that differs from other forms of cell death, such as apoptosis, necrosis, and autophagic cell death in both morphology and biochemical events (2, 3, 4). Ferroptosis has been implicated in diverse pathophysiological processes, including cancer, neurodegeneration, ischemia–reperfusion injury, and tissue inflammation (3, 4). A central biochemical hallmark of ferroptosis is the peroxidation of polyunsaturated fatty acid (PUFA)-containing phospholipids in cellular membranes, a process that ultimately compromises membrane integrity and cellular viability (2, 5, 6) (Fig. 1). Peroxidation of phospholipids in ferroptosis can proceed through both non-enzymatic radical chain reactions driven by iron-dependent Fenton reaction and enzymatic pathways mediated by lipoxygenases, with their relative contributions depending on cellular and metabolic context (3, 5). When the accumulation of lipid peroxides exceeds the cellular capacity for detoxification, ferroptosis is triggered, most prominently through inhibition or loss of the phospholipid hydroperoxide-reducing enzyme glutathione peroxidase 4 (GPX4) or depletion of glutathione (3, 5) (Fig. 1). In addition to GPX4, parallel defense systems, including the ferroptosis suppressor protein-1 (FSP1)–co-enzyme Q10 (CoQ10) axis, have been shown to suppress lipid peroxidation and ferroptosis (7, 8), highlighting the existence of multiple protective mechanisms acting at different steps of lipid peroxidation.

**Fig. 1:**
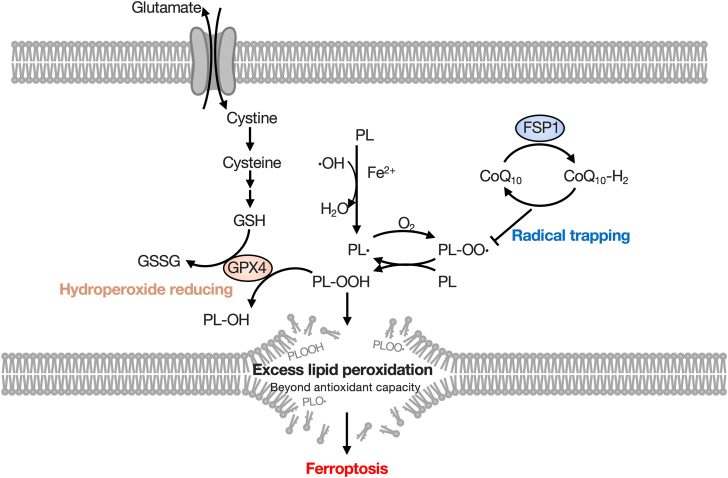
Lipid peroxidation and protective pathways in ferroptosis. Lipid peroxidation is initiated by the abstraction of a hydrogen atom from PUFA-PLs, leading to the formation of a phospholipid radical (PL ˙). PL ˙ subsequently reacts with molecular oxygen to generate a phospholipid peroxyl radical (PLOO ˙). PLOO ˙ then abstracts a hydrogen atom from neighboring PLs, producing phospholipid hydroperoxides (PLOOH) and propagating the chain reaction. Cells possess defense mechanisms—primarily the glutathione (GSH)-dependent reduction of PLOOH by GPX4 and radical trapping by FSP1—to limit lipid peroxidation. However, when these protective systems are impaired, the chain reaction proceeds unchecked, resulting in the accumulation of PLOO ˙ and PLOOH. This accumulation ultimately leads to disruption of membrane integrity and rupture of the cell membrane.

Over the past decade, genetic and chemical screens for ferroptosis sensitivity have uncovered a strong enrichment of genes involved in lipid metabolism. Among these, enzymes responsible for phospholipid acyl chain remodeling have emerged as critical determinants of ferroptotic susceptibility (9, 10). Phospholipid remodeling involves the sequential deacylation and reacylation of phospholipids, thereby reshaping membrane acyl chain composition (11). Consistent with this notion, mass spectrometry-based lipidomic studies have also identified several phospholipid species that either promote or protect against ferroptosis (6, 12, 13). These findings highlight that fine-tuning of membrane phospholipid composition through phospholipid acyl chain remodeling is a key determinant of ferroptosis sensitivity.

In this review, we summarize current knowledge regarding phospholipid remodeling pathways and the specific phospholipid species implicated in ferroptosis, and discuss how these pathways shape membrane lipid composition and influence ferroptosis sensitivity.

## Enzymes involved in phospholipid remodeling

**Fig. 2:**
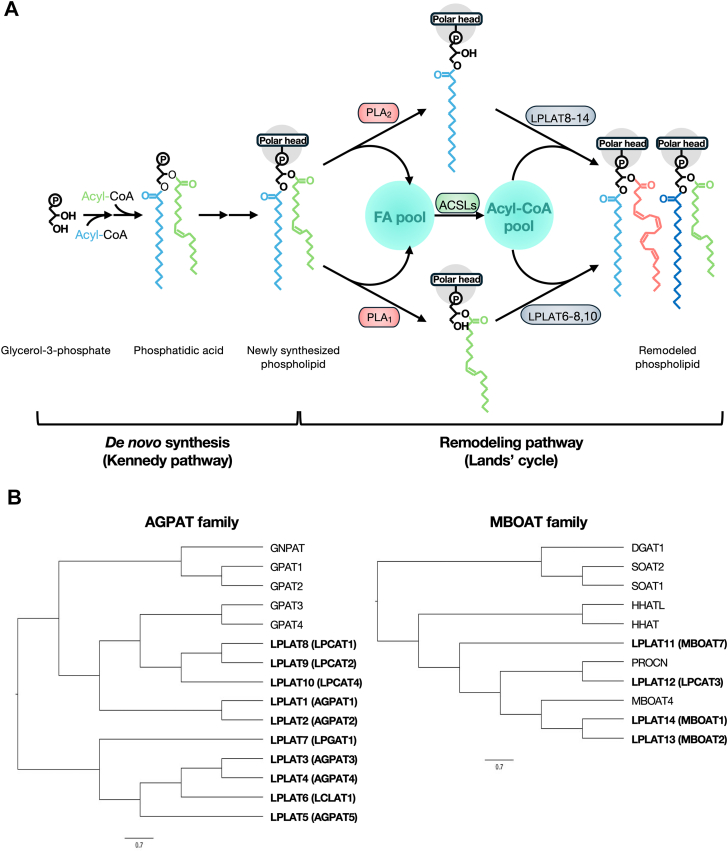
Enzymes involved in phospholipid remodeling. A: De novo synthesis and subsequent acyl chain remodeling of phospholipids. Remodeling at both the *sn-*1 and *sn-*2 positions can occur, thereby increasing fatty acyl diversity in membrane phospholipids. B: Phylogenetic trees of 15 AGPAT family members and 11 MBOAT family members.

Membrane phospholipids exhibit extensive molecular diversity arising from variations in both fatty acyl chains and head groups (14). Based on the specific polar head group attached to the phosphate moiety, phospholipids are categorized into distinct classes, including phosphatidylcholine (PC), phosphatidylethanolamine (PE), phosphatidylserine (PS), phosphatidylinositol (PI), phosphatidylglycerol, and cardiolipin. While each phospholipid class displays characteristic acyl chain compositions, such diversity cannot be fully explained by de novo synthesis alone, in which phospholipid classes are synthesized from a common precursor, phosphatidic acid (PA) (Fig. 2). Instead, this complexity is largely established through the phospholipid remodeling pathway, also known as the Lands’ cycle, following de novo synthesis (11). In this pathway, phospholipases A (PLAs) remove fatty acyl chains from diacyl-phospholipids to produce lysophospholipids (LPLs), which are subsequently reacylated by lysophospholipid acyltransferases (LPLATs) using acyl-CoA substrates (Fig. 2). Through repeated cycles of deacylation and reacylation, cells selectively enrich specific fatty acid species within particular phospholipid classes. Consequently, phospholipid remodeling influences a wide range of membrane properties, including membrane fluidity, curvature, cellular signaling, and susceptibility to peroxidation. Certain types of LPLATs and acyl-CoA synthetases (ACSLs) preferentially utilize PUFAs, while others favor saturated and monounsaturated fatty acids. Accordingly, remodeling pathways that involve such enzymes enhancing or suppressing PUFA incorporation into phospholipids are critical determinants of ferroptosis sensitivity. The enzyme families that mediate phospholipid remodeling—including LPLATs, PLAs, and ACSLs—are discussed in detail in the following sections.

## LPLATs

LPLATs have two substrates, LPLs and acyl-CoAs, and transfer an acyl chain from acyl-CoAs to LPLs, catalyzing the reacylation of LPLs. In mammals, LPLATs are structurally classified into the 1-acylglycerol-3-phosphate *O*-acyltransferase (AGPAT) and the membrane-bound *O*-acyltransferase (MBOAT) families (11) (Table 1). Recently, a systematic nomenclature for LPLATs has been proposed (11), enabling unified classification across enzyme families. It should be noted that some members of these families do not participate in phospholipid remodeling but in other lipid metabolic pathways, including de novo glycerophospholipid synthesis (GPAT1-4, GNPAT, and AGPAT1–5), neutral lipid synthesis (SOAT1, SOAT2, and DGAT1), and protein acylation (HHAT, HHATL, PORCN, and MBOAT4).

**Table 1:** Characteristics of LPLATs involved in phospholipid remodeling

Family	Enzyme	Also Known as	Subcellular localization^a^	*sn*-position Specificity	Lysophospholipid Substrate	Preferred Acyl-CoA	Refs
AGPAT	LPLAT6	LCLAT1, LYCAT	ER	*sn-*1	LPI	C18:0	(30, 31)
	LPLAT7	LPGAT1	ER	*sn-*1	LPC, LPE	C18:0	(32, 33, 34)
	LPLAT8	LPCAT1	ER, PM, LD	*sn-*1	LPC	C16:0, C18:0, C18:1	(35)
				*sn-*2	LPC	C16:0	(26, 27, 28)
	LPLAT9	LPCAT2	ER, LD	*sn-*2	LPC	C20:4 n-6	(24, 25, 26)
	LPLAT10	LPCAT4	ER	*sn-*1	LPC, LPE^b^	C16:0, C18:0, C18:1	(36)
				*sn-*2	LPC, LPE^b^ (LPG)	C16:0, C18:0, C18:1, C20:4 n-6, C22:6 n-3	(36, 98, 99)
MBOAT	LPLAT11	LPIAT1, MBOAT7	ER, MAM	*sn-*2	LPI	C20:4 n-6	(15, 16, 17, 18)
	LPLAT12	LPCAT3, MBOAT5	ER	*sn-*2	LPC, LPE, LysoPS	C18:2 n-6, C20:4 n-6	(18, 19, 20, 21, 22, 23)
	LPLAT13	MBOAT2	ER	*sn-*2	LPC, LPE	C18:1	(18, 19)
	LPLAT14	MBOAT1	ER	*sn-*2	LPE, LysoPS	C18:1	(18, 19)

a ER, endoplasmic reticulum; PM, plasma membrane; LD, lipid droplet; MAM, mitochondria-associated membrane.

b LPLAT10 also utilizes dimethyl- and monomethyl- LPE as substrates.

LPLAT8-14 catalyze a reaction to incorporate a fatty acid into the *sn-*2 position. In general, the *sn-*2 position of phospholipids is occupied by unsaturated fatty acids and represents the major site of PUFA incorporation. Consistent with this, many of these enzymes preferentially utilize unsaturated acyl-CoAs. Among them, LPLAT11 and LPLAT12 (also known as MBOAT7 and LPCAT3, respectively) are responsible for the incorporation of arachidonic acid (C20:4) into PI and PC/PE (15, 16, 17, 18, 19, 20, 21, 22, 23), respectively, whereas LPLAT12 can also utilize linoleoyl (C18:2)-CoA (18, 19, 20, 21). LPLAT13 and LPLAT14 (also known as MBOAT2 and MBOAT1, respectively) preferentially incorporate monounsaturated fatty acids (MUFAs), particularly oleic acid (C18:1), into PC/PE and PE/PS, respectively (18, 19). LPLAT9, originally identified as an enzyme involved in platelet-activating factor (PAF) synthesis (24, 25), has also been reported to incorporate C20:4 into PC (24, 25, 26). In contrast, LPLAT8 (also known as LPCAT1) exhibits activity toward saturated acyl-CoAs and is responsible for the production of di-palmitoyl (C16:0)-PC (26, 27, 28).

Although much of the research on phospholipid remodeling has focused on the *sn-*2 position, remodeling at the *sn-*1 position can also occur (29). To date, four LPLATs—LPLAT6, LPLAT7, LPLAT8, and LPLAT10—have been demonstrated to catalyze fatty acid incorporation at the *sn*-1 position. LPLAT6 (also known as LCLAT1 and LYCAT) incorporates stearic acid (C18:0) into PI at the *sn*-1 position (30, 31). LPLAT7 (also known as LPGAT1) mainly incorporates C18:0 into PC and PE at the *sn*-1 position (32, 33, 34). In addition to the aforementioned activity of introducing C16:0 at the *sn*-2 position, LPLAT8 also introduces C16:0 at the *sn*-1 position (35). This provides a good explanation for why LPLAT8 produces di-palmitoyl-PC in the lung (26, 27, 28). Although less abundant, the latest LC-MS/MS technologies for analyzing fatty acids at the *sn*-1 and *sn*-2 positions have revealed the presence of atypical phospholipid species containing unsaturated fatty acids at the *sn*-1 position. LPLAT10 (also known as LPEAT2 and LPCAT4), a recently reported LPLAT with a unique activity to incorporate unsaturated fatty acids such as C22:6 into the *sn*-1 position of PC/PE, is a responsible LPLAT for such atypical phospholipid species (36).

## PLAs

In phospholipid remodeling, the generation of LPLs by PLAs, upstream of LPLATs, is a critical step that enables selective replacement of fatty acyl chains and enrichment of specific phospholipid species (Fig. 2). However, despite the identification of numerous enzymes with PLA_1_ or PLA_2_ activity (37, 38), the molecular entities responsible for supplying LPL substrates for remodeling remain poorly defined. To date, DDHD1 (also known as PA-PLA_1_ or iPLA1α) has been suggested to function as a remodeling-associated PLA_1_ (39, 40). *ipla-1, a Caenorhabiditis elegans* homolog of DDHD1, has been shown to function as a PI-PLA_1_ acting upstream of the LPLAT6 homologs, *acl-8*, *acl-9*, and *acl-10* (30). Consistent with this, DDHD1 has been implicated in determining the fatty acid composition of PI (41, 42, 43). iPLA2β (also known as PLA2G6), a member of the Ca^2+^-independent PLA_2_ family, may contribute to phospholipid remodeling by supplying LPLs (44, 45); however, its primary role appears to lie in membrane homeostasis and phospholipid turnover rather than in determining phospholipid acyl-chain composition (45, 46, 47).

## ACSLs

ACSLs catalyze the activation of fatty acids into acyl-CoA derivatives, thereby supplying substrates for many fatty acid-utilizing reactions including LPLAT-mediated phospholipid remodeling (Fig. 2). In mammals, the ACSL family comprises five isoforms (ACSL1, ACSL3, ACSL4, ACSL5, and ACSL6) (48). These isoforms differ in their substrate preferences, which shape the composition of intracellular fatty acyl-CoA pools (Table 2). Among them, ACSL4 exhibits high catalytic efficiency and selectivity toward C20:4 (10, 49). ACSL6, in contrast, shows a marked preference for C22:6 (50, 51). ACSL3, although its substrate specificity is less clearly defined in vitro, has been suggested to preferentially activate saturated and monounsaturated fatty acids in cellular contexts (52, 53, 54). On the other hand, ACSL1 and ACSL5 exhibit broader substrate specificities, accepting a wide range of long-chain fatty acids (55, 56, 57). It has been proposed that ACSL interacts with LPLAT to facilitate phospholipid remodeling (58, 59). Recently, MMD (Monocyte-to-Macrophage Differentiation-associated) has been reported to act as a scaffold that interacts with both ACSL4 and LPLAT11, thereby promoting the incorporation of C20:4 into PI (60).

**Table 2:** Characteristics of ACSLs

Enzyme	Preferred Fatty Acids	Subcellular localization^a^	References
ACSL1	C16:0, C18:1, C18:2	ER, MAM, PM	(55, 56, 100, 102)
ACSL3	C16:0, C16:1, C18:1	ER, LD	(52, 53, 54, 101)
ACSL4	C20:4, C20:5	ER, MAM, peroxisomes	(10, 49, 100, 102)
ACSL5	C16:0, C16:1, C18:1, C18:2	ER, MAM, PM, Mito	(56, 57, 100, 102)
ACSL6	C18:2, C18:3 (V1), C22:4, C22:5, C22:6 (V2)	ER, PM	(50, 51, 103, 104)

a Mito, mitochondria.

This scaffolding mechanism underscores a broader paradigm in phospholipid remodeling: the in vivo functions and substrate repertoires of these enzymes may be far more dynamic and extensive than those characterized by in vitro biochemical assays. Rather than relying solely on the intrinsic substrate affinities of isolated enzymes, cellular phospholipid remodeling is likely governed by spatial compartmentalization, protein–protein interactions, and metabolic substrate channeling. For instance, the physical coupling of enzymes enables the efficient transfer of intermediates directly between catalytic sites, thereby shaping specific local phospholipid pools. Furthermore, the distinctive subcellular localization of these enzymes—including their enrichment at specific membrane microdomains or inter-organelle contact sites—may determine their access to distinct substrates. Thus, the protein–protein interactions and spatial context of remodeling enzymes likely play an equally vital role in defining the cellular membrane phospholipid landscape.

## Phospholipid remodeling and ferroptosis

**Fig. 3:**
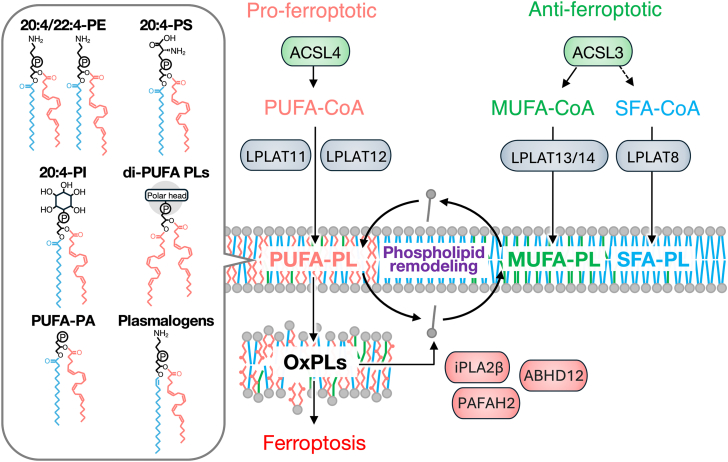
Phospholipid remodeling in Ferroptosis. Phospholipid remodeling determines cellular sensitivity to ferroptosis by regulating the balance between PUFA-containing and MUFA-/SFA-containing phospholipids in cellular membranes. Certain PLAs suppress ferroptosis by removing oxidized fatty acids from phospholipids and the resulting lysophospholipids can be reacylated through the remodeling pathway. Abbreviations: PUFA, polyunsaturated fatty acid; MUFA, monounsaturated fatty acid; SFA, saturated fatty acid.

Phospholipid remodeling enzymes modulate cellular susceptibility to ferroptosis by controlling the incorporation of PUFAs, MUFAs, or saturated fatty acids (SFAs) into membrane phospholipids, as well as by regulating the turnover of oxidized phospholipids (Fig. 3). Below, we systematically summarize phospholipid remodeling enzymes that either promote or suppress ferroptotic cell death.

### Pro-ferroptotic enzymes

ACSL4 is one of the best-established regulators of ferroptosis. ACSL4 catalyzes the activation of long-chain PUFAs into their corresponding acyl-CoA derivatives (49), thereby enabling their incorporation into membrane phospholipids. This process promotes the accumulation of PUFA-containing phospholipids, which serve as substrates for lipid peroxidation. ACSL4 was initially identified as a critical determinant of ferroptosis sensitivity through genetic screening (9, 10, 61). Indeed, genetic deletion or pharmacological inhibition of ACSL4 confers strong resistance to ferroptotic stimuli, shifting the fatty acid composition in membrane phospholipids from PUFA-enriched species to MUFA-containing species (6, 10). Consistent with this, cellular ACSL4 expression levels positively correlate with sensitivity to ferroptosis induced by GPX4 inhibition (10, 61, 62, 63). In addition, accumulating evidence suggests that ACSL4 expression—and consequently cellular susceptibility to ferroptosis—can be dynamically regulated by diverse signaling pathways, including those downstream of cell–cell and cell–extracellular matrix adhesions (64, 65). Notably, ACSL4 is more critical for ferroptosis triggered by direct GPX4 inhibition than for that induced by cystine deprivation (66), suggesting that ferroptosis sensitivity may not be determined solely by the overall abundance of PUFA-containing phospholipids but also by specific phospholipid species and their subcellular localization.

Genome-wide screening also identified LPLAT12 as another key driver of ferroptosis (9). In cooperation with ACSL4, LPLAT12 incorporates C20:4 and adrenic acid (C22:4) into PE, and the resulting PE species containing C20:4 and C22:4 are considered major substrates for lipid peroxidation during ferroptosis (6, 9). However, the ferroptosis resistance conferred by LPLAT12 deficiency was modest compared with the robust protection observed in ACSL4-deficient cells (10, 67), indicating that other LPLATs may also contribute to this process. In line with this, LPLAT11 has emerged as another pro-ferroptotic enzyme. Recent work has demonstrated that LPLAT11 contributes to ferroptosis susceptibility in cancer cells by promoting the accumulation of C20:4-containing PI (C20:4-PI) species (60). This study further showed that the scaffold protein MMD enhances ferroptosis sensitivity through LPLAT11-dependent PI remodeling. Notably, lipidomic analyses in MMD-deficient cells revealed that lipid alterations were not restricted to PI, as decreases in other PUFA-containing PE and PC species were also observed. These findings suggest that LPLAT11-dependent PI remodeling may indirectly expand the cellular pool of peroxidation-susceptible phospholipids. In this context, LPLAT11 may function in parallel with LPLAT12 to promote the synthesis of PUFA-containing phospholipids downstream of ACSL4.

### Anti-ferroptotic enzymes

In contrast to ACSL4, ACSL3 has been reported to suppress ferroptosis. ACSL3 preferentially activates MUFAs to generate MUFA-CoA species (52, 53, 54), which are subsequently incorporated into membrane phospholipids. Through this process, MUFAs can displace PUFAs from membrane phospholipids, thereby reducing the availability of peroxidation-prone lipid substrates. Consistent with this mechanism, several studies have demonstrated that exogenous MUFA supplementation or increased endogenous MUFA production mediated by stearoyl-CoA desaturase 1 (SCD1) suppresses ferroptosis (5, 53, 68, 69). In addition, enrichment of phospholipids with MUFA has been shown to promote ferroptosis resistance in melanoma cells during lymphatic metastasis in an ACSL3-dependent manner (70). Collectively, these findings support a model in which ACSL3-mediated incorporation of MUFAs into membrane phospholipids protects cells from ferroptosis by limiting lipid peroxidation. This process is likely coordinated with downstream LPLATs that incorporate MUFA-CoA species into phospholipids during phospholipid remodeling.

Two LPLATs, LPLAT13 and LPLAT14, have been identified as suppressors of ferroptosis acting downstream of hormone receptor signaling pathways (12). Genetic ablation of either enzyme increased lipid peroxidation and sensitized cells to ferroptosis, whereas their expression promoted resistance to ferroptotic stimuli. Lipidomic analyses revealed that this protective effect is associated with increased incorporation of MUFAs into membrane phospholipids. Consistent with biochemical studies showing that LPLAT13 and LPLAT14 can utilize oleoyl-CoA as an acyl-donor substrate (19), these findings suggest that these enzymes cooperate with MUFA-activating enzymes such as ACSL3 to incorporate MUFAs into phospholipids, thereby limiting the accumulation of peroxidation-susceptible PUFA-containing phospholipid species (Fig. 3).

Increasing phospholipid saturation through the incorporation of SFAs has also been implicated in ferroptosis resistance (71). LPLAT8 promotes the incorporation of saturated acyl chains into membrane phospholipids via the Lands’ cycle, thereby reducing the abundance of PUFA-containing species and limiting lipid peroxidation. In line with this mechanism, LPLAT8 upregulation confers ferroptosis resistance and is associated with increased tumorigenic potential in vivo, whereas its inhibition sensitizes cells to ferroptosis and suppresses tumor growth.

In addition to the displacement of PUFAs from membrane phospholipids by MUFAs and SFAs, the elimination of oxidized PUFAs from phospholipids represents another mechanism that suppresses ferroptosis. Several studies have demonstrated that iPLA2β acts as an anti-ferroptotic regulator by selectively eliminating oxidized PUFA-containing phospholipids, including hydroperoxyeicosatetraenoic acid-PE (HpETE–PE), a key pro-ferroptotic lipid species (72, 73, 74). Through the hydrolytic removal of oxidized acyl chains from membrane phospholipids, iPLA2β limits the accumulation of lipid peroxides and thereby suppresses ferroptotic cell death. Accordingly, depletion of iPLA2β results in increased lipid peroxidation and enhanced ferroptosis sensitivity (74).

PAFAH2 (platelet-activating factor acetylhydrolase 2) is a serine hydrolase that preferentially hydrolyzes oxidized phospholipids (75, 76, 77, 78). This enzyme cleaves oxidized acyl chains from membrane phospholipids to generate lysophospholipids and oxidized fatty acids. Recent studies have demonstrated that PAFAH2 functions as an anti-ferroptotic regulator by detoxifying oxidized phospholipids that accumulate during ferroptosis (79, 80). Genetic ablation or pharmacological inhibition of PAFAH2 sensitizes cells to ferroptosis and promotes the accumulation of ferroptosis-associated lipid species, whereas PAFAH2 overexpression suppresses lipid peroxidation and ferroptotic cell death. In vivo, PAFAH2 deficiency augmented synchronized ferroptosis in renal tubules and exacerbated kidney injury following ischemia/reperfusion. These findings suggest that PAFAH2 contributes to ferroptosis resistance by removing oxidized phospholipids from cellular membranes and thereby limiting the propagation of lipid peroxidation.

Another lipid-metabolizing enzyme implicated in ferroptosis suppression is ABHD12 (α/β-hydrolase domain-containing protein 12), a serine hydrolase involved in lysoPS, oxidized PS, and monoacylglycerol metabolism (81, 82, 83). Chemical proteomic studies identified ABHD12 as a negative regulator of ferroptosis, and pharmacological inhibition of ABHD12 was shown to sensitize cells to ferroptotic cell death (84). Perturbation of ABHD12 activity increased the levels of lysoPS, C20:4-containing PS (C20:4-PS), and 2-arachidonoyl glycerol and promotes ferroptotic cell death. It has been reported that ABHD12 suppresses LPLAT12-mediated incorporation of C20:4 into lysoPS by degrading lysoPS (85). Therefore, it is likely that ABHD suppresses ferroptosis by reducing C20:4-PS levels and degrading oxidized PS.

## Phospholipid species that promote ferroptosis

Accumulating evidence from mass spectrometry-based lipidomics has revealed that diverse phospholipid species are differentially associated with ferroptosis. Notably, specific phospholipid molecular species have been identified as key contributors to ferroptotic cell death (Fig. 3). In this section, we summarize these phospholipid species and their roles in ferroptosis.

### C20:4 and C22:4-containing phospholipids

Among PUFA-containing phospholipids, those containing C20:4 or C22:4 have been most extensively characterized as pro-ferroptotic species. In particular, PE species containing C20:4 or C22:4 were among the first phospholipids identified as major substrates for lipid peroxidation, and their oxidized products are key executioner lipids in ferroptosis (3, 6, 10). The levels of these species are tightly regulated by ACSL4 and LPLAT12. Although C20:4-PE has been the primary focus, other phospholipid classes containing C20:4 also contribute to ferroptosis. C20:4-PI has been implicated in ferroptotic responses in ovarian and renal carcinoma cells in association with LPLAT11 and MMD (60). Furthermore, the levels of C20:4-PS species have also been implicated in ferroptotic responses in ABHD12-inhibited or knockout cells (84). Collectively, these findings highlight that multiple classes of C20:4 and C22:4-containing phospholipids contribute to ferroptosis.

### Di-PUFA phospholipids

Phospholipids containing two PUFA chains (di-PUFA phospholipids) have been identified as potent drivers of ferroptosis (13, 86, 87). Lipidomic analyses across multiple cell lines revealed that the basal levels of di-PUFA PC strongly correlate with cellular sensitivity to ferroptosis (13). Consistently, exogenous supplementation of di-PUFA phospholipids, including both di-PUFA PC and di-PUFA PE, enhances ferroptosis, supporting their roles in ferroptosis susceptibility (13, 87). Mechanistically, di-PUFA species are more susceptible to peroxidation than mono-PUFA species, consistent with their higher density of bis-allylic hydrogen atoms and enhanced capacity to propagate lipid peroxidation chain reactions (87). Beyond their intrinsic susceptibility to peroxidation, di-PUFA PCs have been reported to interact with the mitochondrial complex I, thereby promoting reactive oxygen species (ROS) generation (13). While these findings firmly link di-PUFA phospholipids to ferroptosis, establishing a definitive causal role remains challenging because exogenous lipid supplementation may not fully recapitulate endogenous lipid metabolism, trafficking, or membrane distribution. Thus, precise genetic or chemical strategies to modulate endogenous di-PUFA phospholipids are warranted to fully substantiate their contributions.

### Phosphatidic acid (PA)

Phosphatidic acid (PA) has emerged as a mediator of a noncanonical ferroptosis pathway independent of ACSL4, GPX4, and oxidized PE species. It has been shown that ferroptotic cell death can arise in vivo under conditions of high oxidative stress, even in the absence of exogenous ferroptosis inducers, and involves p53 activation and downstream effectors such as pleckstrin homology-like domain family A member 2 (PHLDA2) and 12-lipoxygenase (73, 88, 89, 90). In this context, ROS-driven peroxidation of PA has been identified as a key lipid event associated with ferroptosis (89). Mechanistically, this pathway operates independently of canonical PUFA-containing phospholipids, suggesting that PA can serve as an alternative substrate for lipid peroxidation (89). Furthermore, recent work has demonstrated that GPX1 suppresses this pathway through its association with the endoplasmic reticulum (ER)-resident lipid transfer protein OSBPL8 (oxysterol binding protein-like 8), thereby reducing oxidized PA in the ER (90). Although GPX1 alone cannot reduce phospholipid hydroperoxides (90, 91), it reduces PA hydroperoxides in the presence of OSBPL8 in an in vitro biochemical assay (90). Thus, PA represents an alternative lipid axis driving ferroptosis.

### Plasmalogens

Peroxisomes play a critical role in the biosynthesis of ether-linked phospholipids, including plasmalogens, which have been implicated in ferroptosis. These lipids are characterized by a vinyl ether bond at the *sn*-1 position, which is particularly susceptible to oxidative attack. This structural feature originates from a distinct peroxisomal biosynthetic pathway (92), highlighting an alternative route for generating peroxidation-prone lipids. Consistently, modulation of ether lipid biosynthesis alters cellular sensitivity to ferroptosis, with reduced synthesis conferring resistance and increased production promoting ferroptotic cell death (93, 94). Although plasmalogens have been proposed to exhibit antioxidant properties in other biological contexts (92, 95), direct evidence for such a protective role in ferroptosis remains limited, and current data instead support their contribution to lipid peroxidation and ferroptosis.

Collectively, these findings indicate that ferroptosis is governed by a spectrum of phospholipid species with distinct structural features, biosynthetic origins, and susceptibilities to peroxidation. Rather than a single lipid entity, ferroptosis appears to arise from the coordinated contribution of multiple lipid classes across different metabolic pathways.

## Conclusions and future perspectives

In summary, ferroptosis is shaped by phospholipid remodeling pathways that generate and regulate specific phospholipid species susceptible to peroxidation. Consistently, accumulating evidence highlights the involvement of defined classes of phospholipids, including PUFA-containing phospholipids, di-PUFA phospholipids, PA, and ether lipids.

A critical, yet unresolved question is why certain phospholipid species contribute disproportionately to ferroptosis. This phenomenon likely arises from a synergy of factors: the intrinsic chemical susceptibility of these phospholipids to iron-catalyzed peroxidation, their accumulation within peroxidation-vulnerable membrane microdomains, and their effects on additional signaling and biophysical mechanisms. Furthermore, given that ferroptosis is not a uniform global event but rather a spatially orchestrated process (13, 90, 96, 97), these phospholipids may undergo selective transport via inter-organelle lipid trafficking proteins to specific organelle membranes that serve as the initiation sites for ferroptosis.

This specificity raises a broader conceptual question: whether ferroptosis is driven by the selective oxidation of specific phospholipids or by a global shift in membrane oxidative state. It is likely that both models are at work, depending on the cell types. In the former model, downstream effectors that recognize oxidized lipids and execute cell death should exist but remain to be identified. In the latter model, ferroptosis sensitivity may instead be governed by the overall balance of phospholipid remodeling, which varies across cell types and pathological or environmental conditions.

Taken together, context-dependent regulation of phospholipid remodeling likely plays a central role in determining ferroptosis sensitivity. This perspective may provide a unifying framework for understanding ferroptosis across diverse biological settings and could facilitate the development of therapeutic strategies targeting ferroptosis-associated diseases, including cancer, neurodegenerative disorders, and ischemia–reperfusion injury.

## Data availability

All data generated or analyzed during this study are included in this published article and its supporting information files. Raw data is available upon request.

## Conflict of interest

The authors declare that they have no conflicts of interest with the contents of this article.
